# Akt1 deficiency does not affect fiber type composition or mitochondrial protein expression in skeletal muscle of male mice

**DOI:** 10.14814/phy2.70048

**Published:** 2024-09-10

**Authors:** Tatsuya Miyaji, Ryuichi Kasuya, Atsushi Sawada, Daisuke Sawamura, Yu Kitaoka, Mitsunori Miyazaki

**Affiliations:** ^1^ Department of Integrative Physiology, Graduate School of Biomedical and Health Sciences Hiroshima University Higashihiroshima Japan; ^2^ Department of Physical Therapy, School of Rehabilitation Sciences Health Sciences University of Hokkaido Tobetsu Japan; ^3^ Department of Rehabilitation Science, Faculty of Health Sciences Hokkaido University Sapporo Japan; ^4^ Department of Human Sciences Kanagawa University Yokohama Japan

**Keywords:** Akt1, glycolysis, mitochondria, skeletal muscle

## Abstract

Insulin‐like growth factor‐1‐induced activation of ATP citrate lyase (ACLY) improves muscle mitochondrial function through an Akt‐dependent mechanism. In this study, we examined whether Akt1 deficiency alters skeletal muscle fiber type and mitochondrial function by regulating ACLY‐dependent signaling in male Akt1 knockout (KO) mice (12–16 weeks old). Akt1 KO mice exhibited decreased body weight and muscle wet weight, with reduced cross‐sectional areas of slow‐ and fast‐type muscle fibers. Loss of Akt1 did not affect the phosphorylation status of ACLY in skeletal muscle. The skeletal muscle fiber type and expression of mitochondrial oxidative phosphorylation complex proteins were unchanged in Akt1 KO mice compared with the wild‐type control. These observations indicate that Akt1 is important for the regulation of skeletal muscle fiber size, whereas the regulation of muscle fiber type and muscle mitochondrial content occurs independently of Akt1 activity.

## 1 INTRODUCTION

Maintaining skeletal muscle mass and metabolic capacity is necessary to avoid the risk of developing sarcopenia, frailty, and metabolic diseases, thus contributing to a healthy lifestyle. Akt‐dependent regulation is an important signaling pathway that controls skeletal muscle mass and energy metabolism (Huang et al., 2018; Moriya & Miyazaki, 2018; Schiaffino et al., 2013; Whiteman et al., 2002). Activation of the Akt‐dependent pathway stimulates muscle protein synthesis and hypertrophic growth by enhancing protein translation efficiency and ribosomal biogenesis by regulating the mechanistic target of rapamycin complex 1 signaling (Bodine et al., 2001; Marabita et al., 2016; Rommel et al., 2001). Activation of the Akt‐dependent pathway also suppresses the muscle proteolytic system by regulating the transcription factor Forkhead box O (FoxO) family (Sandri et al., 2013). Decreased Akt activity results in dysfunctional glucose transport and blood glucose uptake, particularly in insulin‐sensitive tissues, including skeletal muscle (Chadt & Al‐Hasani, 2020; Jaiswal et al., 2019). There are three distinct Akt isoforms (Akt1, Akt2, and Akt3), each translated from a different gene, but exhibiting high amino acid homology. These three isoforms have overlapping and/or distinct cellular function (Franke, 2008). Akt1 and Akt2 are predominantly expressed in skeletal muscle. Akt1 is implicated in the regulation of skeletal muscle cell growth and muscle satellite cell proliferation, whereas Akt2 is involved in glucose metabolism and insulin‐dependent signaling (Bae et al., 2003; Mackenzie & Elliott, 2014; Moriya & Miyazaki, 2018).

While the activation of Akt‐dependent pathways has a key role in the regulation of skeletal muscle mass/fiber size and glucose metabolism, the role of Akt in oxidative energy metabolism remains largely unknown. Skeletal muscle from mice lacking the skeletal muscle‐specific Akt1/Akt2 gene exhibits significant impairment of oxidative metabolic capacity (Jaiswal et al., 2022). Inactivation of glycogen synthase kinase 3 beta, a direct downstream target of Akt, also promotes oxidative metabolism in skeletal muscle cells through the peroxisome proliferator‐activated receptor gamma coactivator 1‐alpha (PGC‐1α) pathway (Theeuwes et al., 2017). Activation of ATP citrate lyase (ACLY) by upstream insulin‐like growth factor‐1 (IGF‐1) improves skeletal muscle mitochondrial function in an Akt‐dependent manner (Das et al., 2015). These earlier studies suggest that Akt‐dependent pathways may regulate mitochondrial function and oxidative energy metabolism in skeletal muscle. In this study, the role of Akt1 in oxidative energy metabolism in skeletal muscle was examined, with a focus on whether Akt1 deficiency causes muscle fiber type alteration and mitochondrial dysfunction.

## 2 MATERIALS AND METHODS

### 2.1 Antibodies

Monoclonal Anti‐Myosin (Skeletal, Slow, Cat#: M8421), Anti‐Laminin (Cat#: L9393), and PGC‐1 (Cat#: AB3242) were from Merck (Darmstadt, Germany). Akt (pan, Cat#: 4691), Akt1 (Cat#: 2967), Akt2 (Cat#: 2964), ACLY (Cat#: 13390), phospho‐FoxO1 (Thr24)/FoxO3a (Thr32) (Cat#: 9464), and FoxO1 (Cat#: 2880) were from Cell Signaling Technology (Danvers, MA, USA). Total OXPHOS Rodent WB Antibody Cocktail (Cat#: ab110413) was from abcam (Cambridge, UK). phospho‐ACLY (S455, Cat#: AP‐779) was from ABclonal (Wuhan, China). GLUT4 (Cat#: 66846‐1‐Ig) was from Proteintech (Rosemont, IL, USA). Peroxidase AffiniPure Goat Anti‐Rabbit IgG (Cat#: 111‐035‐003) and Anti‐Mouse IgG (Cat#: 115‐035‐003) were from Jackson ImmunoResearch Laboratories (West Grove, PA, USA). Alexa Fluor 488‐conjugated goat anti‐rabbit IgG (Cat#: A‐11008) and Alexa Fluor 594‐conjugated goat anti‐mouse IgG (Cat#: A11005) were from Thermo Fisher Scientific (Waltham, MA, USA).

### 2.2 Animal care and use

All experimental procedures were approved by the Animal Ethics and Research Committee of the Health Sciences University of Hokkaido (no. 19‐060). All animals were kept on a 12‐h light/dark cycle in a temperature‐ and humidity‐controlled room (24°C ± 1°C and 50%–60%). Autoclaved sterile water and certified rodent diet (MF, Oriental Yeast, Tokyo, Japan) were provided ad libitum. The breeding pairs (first generation) of Akt1 heterozygous mice (B6.129P2‐*Akt1* ^*tm1Mbb*^/J) were obtained from The Jackson Laboratory (Bar Harbor, ME, USA). Fifth to seventh‐generation heterozygous mice were allowed to breed to produce Akt1 KO mice. Animals were fed with the regular diet during breeding. Wild‐type (WT) mice from the same breeding colony were used as controls. All mice were male and fully sexually mature (12–16 weeks old). Skeletal muscle samples (gastrocnemius and soleus muscles) were collected under isoflurane inhalation anesthesia (2.0% isoflurane in air), which were weighed, quickly frozen in liquid nitrogen, and stored at −80°C until further analysis. After tissue collection was complete, the mice were euthanized under anesthesia by cervical dislocation.

### 2.3 Protein extraction and western blot analysis

Frozen tissue samples were homogenized in ice‐cold RIPA buffer containing a protease inhibitor cocktail (25955–24; Nacalai Tesqu, Kyoto, Japan). Homogenates were centrifuged at 16,000 × *g* for 10 min at 4°C, and the supernatant was collected as the protein sample. Protein concentration was measured using the Pierce BCA protein assay kit (23225; Thermo Fisher Scientific, Waltham, MA, USA). Protein lysate was mixed with 1/4 volume of 4× SDS‐PAGE sample buffer (196‐16142; FUJIFILM Wako Pure Chemical Corporation, Osaka, Japan) and adjusted to 1.0 μg/μL concentration following overnight incubation at room temperature. Equivalent amounts of protein were separated using a precast polyacrylamide gel system (e‐PAGEL E‐R520L, 5%–20% acrylamide gradient gel; ATTO, Tokyo, Japan or Mini‐PROTEAN TGX Any kD Precast Protein Gels, #4569036, precast polyacrylamide gel for any kD; BIO‐RAD, Hercules, CA, USA) and transferred (for e‐PAGEL; 300 mA constant current, 60–80 min, 4°C cold transfer buffer with ice‐cooling unit, using Criterion Blotter; BIO‐RAD, Hercules, CA, USA, and for TGX gels; 2.5 A constant current, 3 min at room temperature, using Trans‐Blot Turbo System; BIO‐RAD) to polyvinylidene difluoride membranes. The membranes were blocked with Bullet Blocking One for western blotting (13779‐01; Nacalai Tesqu, Kyoto, Japan) and incubated with the appropriate dilutions of the primary (1:2000 dilution for GLUT4 antibody, 1:1000 for all others) and secondary (1:15,000 dilution for both Anti‐Rabbit IgG and Anti‐Mouse IgG) antibodies. Bound antibody complexes were imaged and quantified using chemiluminescence reagents (ImmunoStar Zeta, 291‐72401, or ImmunoStar LD, 296‐69901; FUJIFILM Wako Pure Chemical Corporation, Osaka, Japan), C‐DiGit Blot Scanner and Image Studio Digits 5.2 software (LI‐COR Biosciences, Lincoln, NE, USA). Detailed experimental conditions are shown in Figure S1, with the original Western blotting images.

### 2.4 Immunohistochemistry

Skeletal muscle samples for histochemistry or immunohistochemistry were frozen in isopentane, which was cooled by liquid nitrogen, sliced into 10 μm‐thick sections using a cryostat (Leica CM 1860; Leica Biosystems, Eisfeld, Germany), and stored at −80°C until analysis. Sections were fixed with 4% paraformaldehyde, permeabilized with 0.1% Triton X‐100, and blocked with 1% bovine serum albumin. Monoclonal anti‐myosin (Skeletal, Slow) and Alexa Fluor 594‐conjugated goat anti‐mouse IgG (H + L) antibodies were used for detecting the localization of slow‐MHC. Rabbit anti‐laminin and Alexa Fluor 488‐conjugated goat anti‐rabbit IgG (H + L) antibodies were used for detecting the localization of laminin. Tissue sections were mounted with Vectashield mounting medium containing DAPI (H‐1200; Vector Laboratories, Newark, CA, USA) and fluorescence microscopy images were captured using an Olympus IX73 system with UPLFLN4X and UPLFLN20X as objective lenses and cellSens imaging software (Olympus, Tokyo, Japan). The cross‐sectional area of the muscle fibers was determined by a grid sampling method using immunofluorescence‐stained 20x magnified images and WinROOF image analysis software (Mitani, Tokyo, Japan). Three to five non‐overlapping areas were randomly selected and identically distributed in each whole muscle section, so that a total of 200–300 fibers per muscle sample were analyzed.

### 2.5 PAS staining

To assess glycogen content in skeletal muscle, histochemical analysis of muscle sections with periodic acid‐Schiff (PAS) staining was performed. PAS staining kits (15792; Muto Pure Chemicals, Tokyo, Japan) were used and all procedures were in accordance with the manufacturer's recommendations. Stained images were obtained using a BZ‐X810 (Keyence, Osaka, Japan) with a Plan Apochromat 10X (BZ‐PA10; Keyence, Osaka, Japan) as objective lens.

### 2.6 Enzyme activity

Frozen muscle samples were homogenized in 50 times (volume/weight) of 100 mM potassium phosphate buffer. The maximal enzyme activity of citrate synthase (CS), cytochrome c oxidase (COX), phosphofructokinase (PFK), and lactate dehydrogenase (LDH) was determined by spectrophotometry based on previously established protocols (Kitaoka et al., 2021; Spinazzi et al., 2012).

### 2.7 Statistical analysis

All results are expressed as means ± SD. Comparison between WT and Akt1 KO was done using an unpaired *t*‐test. The statistical significance level for all comparisons was set at *p* < 0.05.

## 3 RESULTS

### 3.1 Decreased body weight and muscle fiber size in Akt1 KO mice

**FIGURE 1:**
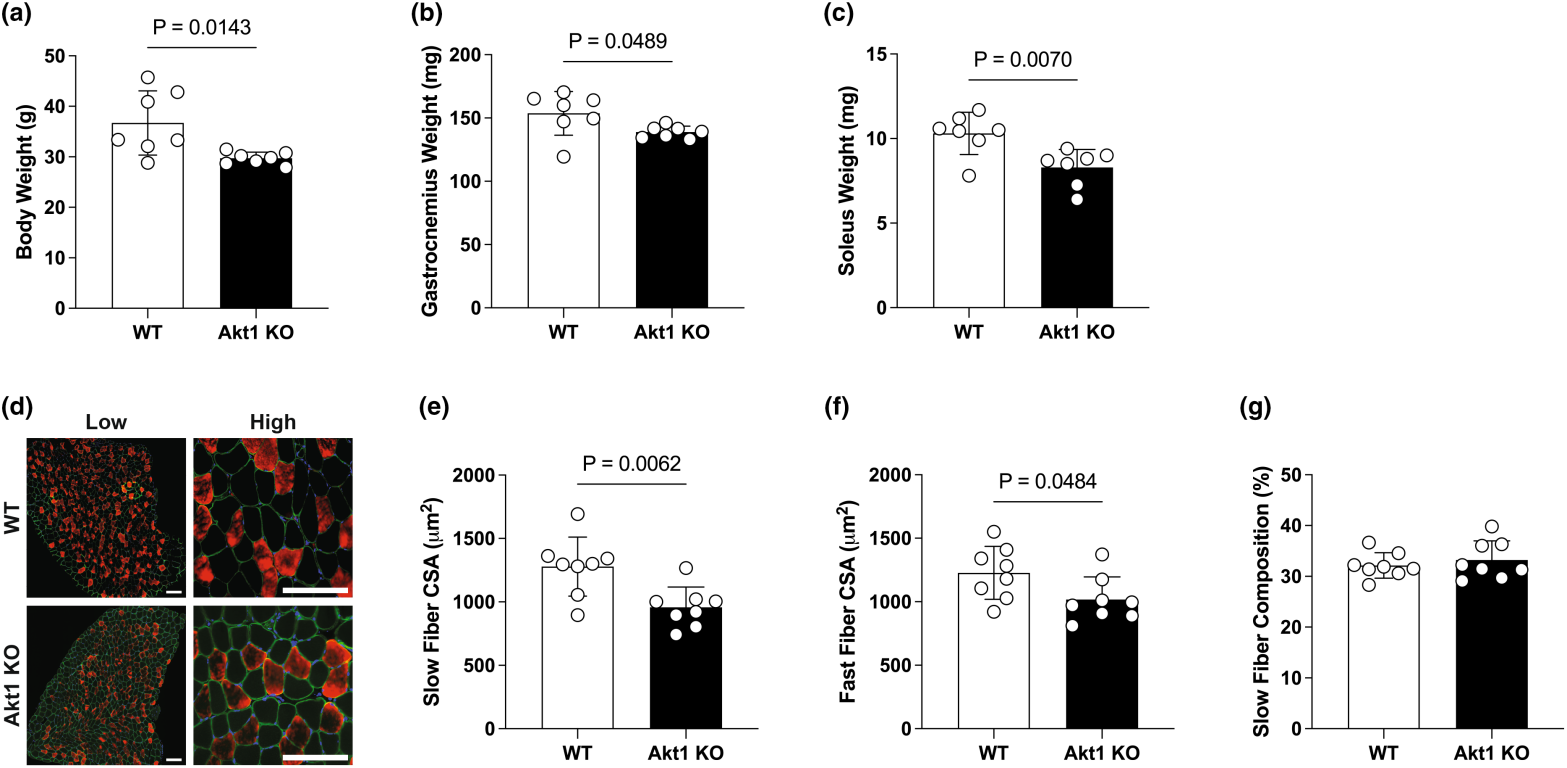
Decreased body weight and muscle fiber size in Akt1 KO mice. (a) Body weight (g). Wet weight (mg) of (b) gastrocnemius muscle, and (c) soleus muscle. *n* = 7 in each group for body weight and muscle weight. (d) Representative immunostaining images of soleus muscle cross‐sections (Blue; 4′,6‐diamidino‐2‐phenylindole, Green; laminin, Red; slow‐myosin heavy chain). Negatively‐stained black fibers were designated as fast‐type fibers. The scale bar shows 100 μm. The mean size of cross‐sectional area in (e) slow‐type and (f) fast‐type fibers. (g) Composition (%) of slow‐type fibers was calculated by counting a total of 200–300 fibers from different regions in each sample. *n* = 8 in each group for muscle fiber size analysis. All results were expressed as mean ± SD. The *p*‐values are indicated directly in the figure for significant difference.

Compared with the wild‐type control, Akt1 KO mice exhibited significantly decreased body weight (19.09% decrease; WT, 36.72 ± 6.35 g; Akt1 KO, 29.71 ± 1.23 g; Figure 1a) and muscle wet weight (9.64% decrease in fast‐type predominant gastrocnemius muscle: WT, 153.71 ± 17.28 mg; Akt1 KO, 138.89 ± 4.67 mg; Figure 1b and 19.54% decrease in soleus muscle, which is a mixture of slow and fast‐type muscles: WT, 10.31 ± 1.25 mg; Akt1 KO, 8.29 ± 1.07 mg; Figure 1c). The muscle fiber size in the soleus muscle was significantly smaller for the slow (25.15% decrease; WT, 1278.48 ± 232.44 μm^2^; Akt1 KO, 956.97 ± 160.17 μm^2^; Figure 1e) and fast muscle fibers (17.14% decrease; WT, 1227.10 ± 208.40 μm^2^; Akt1 KO, 1016.72 ± 179.64 μm^2^; Figure 1f). No significant differences were observed in fiber type composition (percentage of slow‐type muscle fiber) in the soleus muscle between the WT and Akt1 KO mice (WT, 32.14 ± 2.51%; Akt1 KO, 33.24 ± 3.73%; Figure 1g). The results indicate that Akt1 deficiency results in decreased muscle fiber size, but does not affect muscle fiber type composition.

### 3.2 Akt1 deficiency does not affect mitochondrial oxidative energy metabolism in skeletal muscle

**FIGURE 2:**
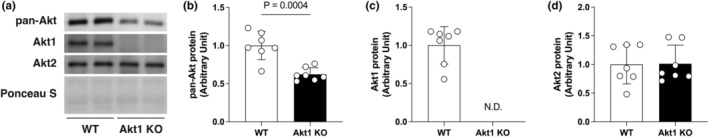
Protein expression of Akt isoforms in Akt1 KO mice. (a) Representative images of Western blotting. Equal amounts of protein loading were confirmed by Ponceau S staining. Relative protein expression of Akt isoforms in the soleus muscle of WT and Akt1 KO mice were quantified. (b) pan‐Akt; (c) Akt1; (d) Akt2. Akt1 protein was not detected (N.D.) in Akt1 KO. *n* = 7 in each group. All results were expressed as mean ± SD. The *p*‐values are indicated directly in the figure for significant difference.

**FIGURE 3:**
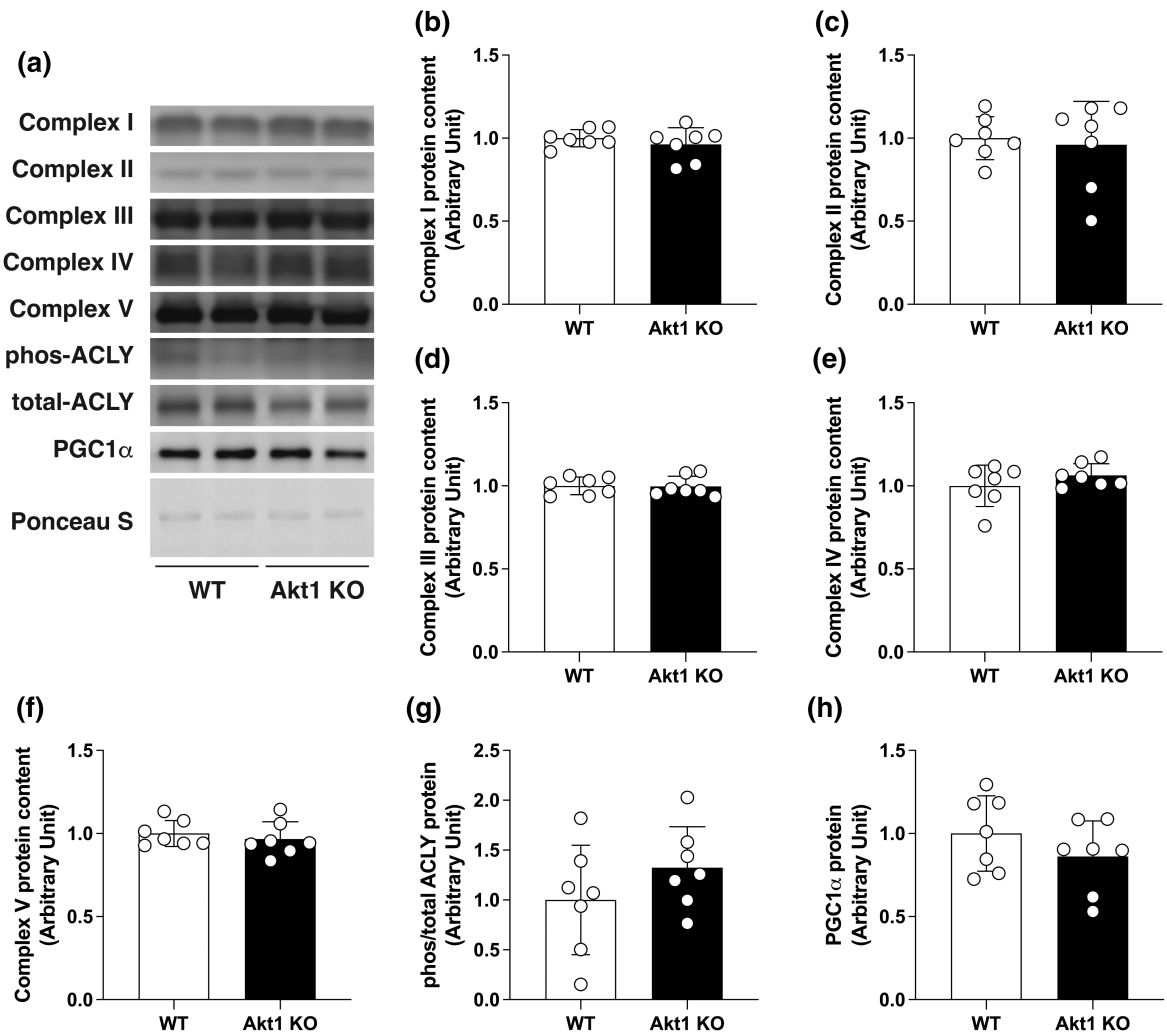
Akt1 deficiency did not affect mitochondrial oxidative energy metabolism in skeletal muscle. (a) Representative images of western blotting. Equal amounts of protein loading were confirmed by Ponceau S staining. Relative protein expression of OXPHOS protein in the soleus muscle of WT and Akt1 KO mice were quantified. (b–f) complex I to V; (g) phos/total ACLY; (h) PGC1α. Phosphorylation level of ACLY protein was expressed as relative to total protein. *n* = 7 in each group. All results were expressed as mean ± SD.

The expression of pan‐Akt protein in the soleus muscle of Akt1 KO mice was significantly decreased by 37.64% compared with the WT control. No Akt1 protein expression was observed in the skeletal muscle of Akt1 KO mice, whereas Akt2 expression levels were comparable to that of the WT (Figure 2). To determine the muscle mitochondrial content, which has a major impact on oxidative energy metabolism in skeletal muscle, the expression of mitochondrial oxidative phosphorylation (OXPHOS) complex proteins was measured. The expression of OXPHOS protein in the soleus muscle of Akt1 KO mice was not significantly different in any of the complexes from I to V compared with that of the WT. There was no effect of Akt1 deficiency on the phosphorylation status of ACLY. The level of PGC‐1α protein expression, which is a master regulator of mitochondrial biogenesis, was also unchanged by Akt1 deficiency (Figure 3).

### 3.3 Muscle enzymatic activities of the glycolytic system are diminished by Akt1 deficiency

**FIGURE 4:**
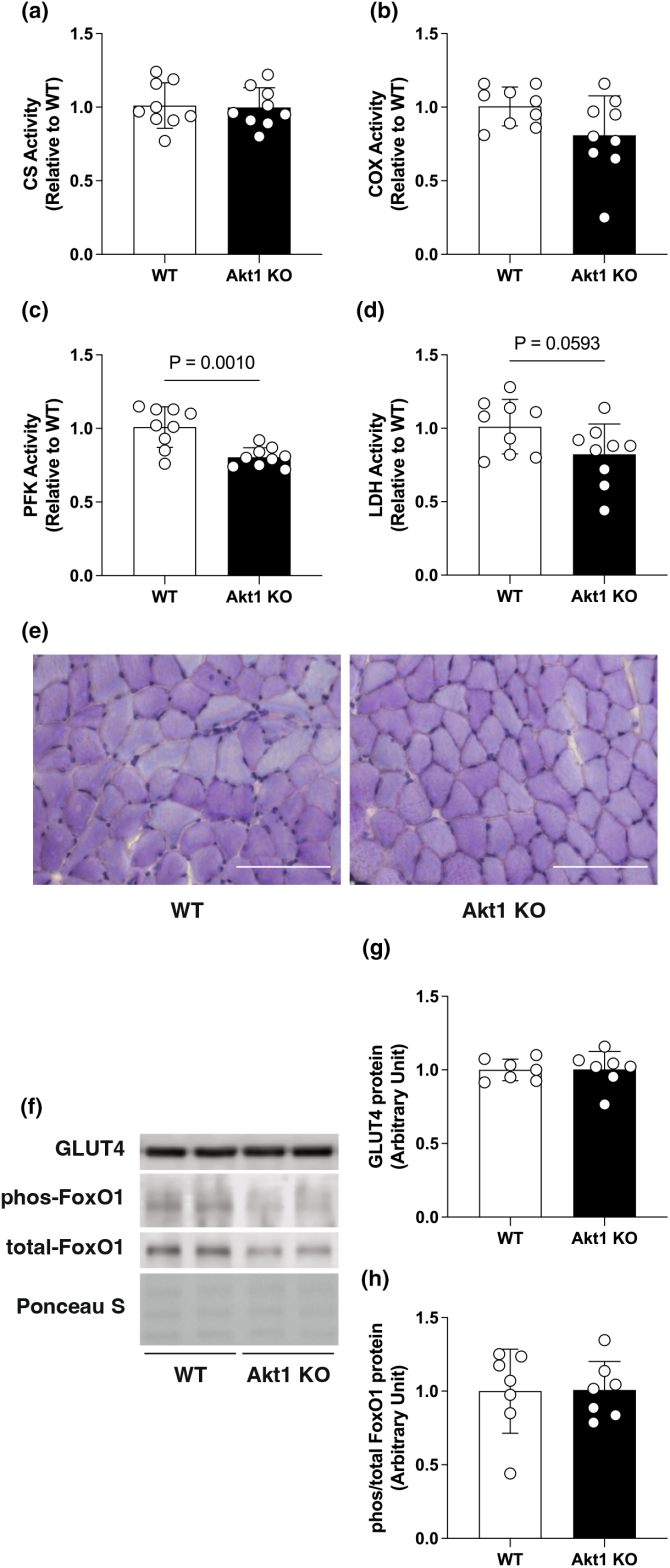
Decreased glycolytic enzyme activity in the soleus muscle of Akt1 KO mice. Oxidative or glycolytic enzyme activities of (a) citrate synthase, (b) cytochrome c oxidase, (c) phosphofructokinase, and (d) lactate dehydrogenase was determined. *n* = 9 in each group for enzyme activity assays. (e) Representative histological images of soleus muscle cross‐sections with PAS staining. The scale bar shows 100 μm. (f) Representative image of Western blotting and quantification for (g) glucose transporter type 4, and (h) forkhead box O 1 (FoxO1) in the soleus muscle of WT and Akt1 KO mice. Equal amounts of protein loading were confirmed by Ponceau S staining. Phosphorylation level of FoxO1 protein was expressed as relative to total protein. *n* = 7 in each group for Western blotting. All results were expressed as mean ± SD. The *p*‐values are indicated directly in the figure.

Similar to the expression of the OXPHOS subunit, CS (a typical hallmark of the tricarboxylic acid cycle and an indicator of mitochondrial abundance, Figure 4a) and COX (a functional indicator of the electron transport chain, Figure 4b) enzyme activities were unaffected by Akt1 deficiency and were comparable to the WT controls. In contrast, the enzymatic activity of PFK, a rate‐limiting enzyme in the glycolytic system, was significantly decreased by 23.12% in the skeletal muscle of Akt1 KO mice (Figure 4c). The activity of LDH, which catalyzes the interconversion of lactate and pyruvate, was likely diminished with Akt1 gene deletion (22.63% decrease in Akt1 KO compared to WT, *p* = 0.059, Figure 4d). Muscle glycogen content was evaluated by PAS staining, and both WT and Akt1 KO showed comparable staining intensity in soleus muscle, with no obvious differences (Figure 4e). The expression of glucose transporter type 4 (GLUT4) protein, a major glucose transporter in skeletal muscle for insulin‐dependent glucose uptake, was unchanged by Akt1 deficiency. The phosphorylation status of FoxO1 was also unaffected in Akt1 KO compared to WT (Figure 4f–h). These results indicate that Akt1 likely has an important role in metabolic regulation of the glycolytic system, but is not involved in regulation of muscle glycogen content at steady state or in mitochondria‐mediated oxidative energy production.

## 4 DISCUSSION

In this study, we determined whether the loss of the Akt1 gene affects skeletal muscle mass/size, as well as mitochondrial volume and oxidative energy metabolism. Consistent with previous studies (Cho, Thorvaldsen, et al., 2001; Miyazaki et al., 2020; Moriya & Miyazaki, 2018), our results indicate that skeletal muscle fiber size in Akt1‐deficient mice was significantly decreased; however, mitochondrial content and oxidative metabolic capacity were unaffected. In contrast, the enzyme activities of the glycolytic system in skeletal muscle were decreased with Akt1 gene deletion, indicating that Akt1 is possibly involved in part of the energy production system through glycogenolysis/glycolysis.

ACLY is a cytosolic enzyme that promotes de novo fatty acid synthesis by converting citric acid into the fatty acid precursor acetyl CoA. (Das et al., 2015) found that IGF‐1/Akt‐dependent ACLY activation in skeletal muscle promotes increased mitochondrial respiratory chain complex protein expression and supercomplex activity by enhancing cardiolipin synthesis, thereby contributing to improved mitochondrial function. However, in the present study, no changes in the indicators of mitochondrial content, including components of both the tricarboxylic acid cycle and electron transport chain, were observed in the skeletal muscle of Akt1 KO mice. This suggests that of the three Akt isoforms, Akt1 may not contribute to the regulation of oxidative metabolic capacity in skeletal muscle. A reasonable interpretation of this observation is that Akt2, which is abundantly expressed in skeletal muscle along with Akt1, may compensate for the molecular function of Akt1 and contribute to the maintenance of mitochondrial function and oxidative capacity in the skeletal muscle of Akt1 KO mice. Because of the 37.64% reduction in pan‐Akt protein expression levels in the skeletal muscle of Akt1 KO mice, and no change in Akt2 protein expression levels, the reduction of pan‐Akt levels is the result of Akt1 deficiency. In the skeletal muscle of Akt1 KO mice, the signal input from the remaining Akt (likely Akt2) may be sufficient for the maintenance of mitochondrial function. Consistent with this idea, the skeletal muscle of the Akt2 KO mouse exhibits decreased mitochondrial DNA and reduced expression of genes associated with mitochondrial biogenesis (Chen et al., 2020). Moreover, different research groups reported that a decreased proportion of slow muscle fibers, abnormal mitochondrial biogenesis, and mitochondrial dysfunction are observed in the skeletal muscle of muscle‐specific Akt1/Akt2 double‐KO mice (Jaiswal et al., 2022; Sasako et al., 2022). Combined with the results of the present study, it is likely that the Akt isoform involved in the regulation of mitochondrial volume and function in skeletal muscle is Akt2, not Akt1.

Despite no changes in the indicators of mitochondrial function or oxidative metabolic capacity, the skeletal muscles of Akt1 KO mice showed a significant decrease in enzyme activity of the glycolytic system. In insulin‐sensitive tissues, including skeletal muscle, blood glucose uptake is mediated by Akt, a downstream signaling cascade of the insulin receptor that mediates the translocation of GLUT4 to the plasma membrane (Chadt & Al‐Hasani, 2020; da Silva Rosa et al., 2020; Jaiswal et al., 2019). Akt2 is particularly important for the regulation of insulin‐dependent glucose uptake, resulting in the induction of insulin resistance and disruption of glucose homeostasis in Akt2 KO mice (Cho, Mu, et al., 2001), whereas Akt1 is essential for normal cell growth, but dispensable for insulin‐regulated glucose metabolism both in male and female Akt1 KO mice (Cho, Thorvaldsen, et al., 2001). Indeed, although GLUT4 translocation was not addressed, our results also showed no significant changes in GLUT4 protein expression in the skeletal muscles of Akt1 KO mice; however, insulin‐independent glycolytic metabolism was found to be partly defective with Akt1 deficiency. It is currently unclear how Akt1 affects glycolytic metabolism in an insulin‐independent manner in skeletal muscle. A previous study using postmortem bovine muscle reported that increased PFK activity and glycolysis are induced following activation of the Akt pathway in response to oxidative stress (Chen et al., 2022). In the skeletal muscle of Akt1 transgenic mice, which express a constitutively active form of Akt1 specifically in skeletal muscle, the expression of glycolytic enzymes, including PFK and LDH, was upregulated (Akasaki et al., 2014). Furthermore, in the skeletal muscle of aged muscle‐specific Akt1/Akt2 double‐KO mice, the expression of genes associated with the glycolytic system, including PFK, and the proportion of glucose‐rich type 2 fibers were decreased (Sasako et al., 2022). Because FoxO1/4 dual ablation improves the defective phenotype of Akt1/Akt2 double‐KO mice, the regulation of glycolytic metabolism by Akt is likely mediated through FoxO regulation (Sasako et al., 2022). However, in the present study, no effect of Akt1 deficiency was observed on the phosphorylation status of FoxO1 in skeletal muscle. These earlier studies and our current data highlight the importance of further investigation into the mechanisms involved in the regulation of glycolytic metabolism in skeletal muscle through Akt1, particularly including the FoxO pathway.

## 5 LIMITATION

This study is partly descriptive and has some clear limitations, including (1) the lack of physiological analysis in Akt1 KO mice of possible abnormalities that could be predicted with impaired glucose metabolism, including blood glucose alterations as well as intolerance to high‐intensity exercise, which is dependent on glycolytic metabolism in skeletal muscle; (2) the absence of oxygen flux analysis to assess skeletal muscle mitochondrial function; (3) due to the potential impact of sex hormones including estrogen on both oxidative and glycolytic energy metabolism in skeletal muscle (Ventura‐Clapier et al., 2019), only male mice were exclusively used in the analysis, thus making it difficult to address possible sex differences; and (4) as the use of the total Akt1 KO model, it is not possible to rule out the potential impact of Akt1 loss in other organ systems.

## AUTHOR CONTRIBUTIONS

MM conceived and designed the project; TM, RK, AS, DS, and YK acquired, analyzed, and interpreted the data; TM and MM wrote, revised, and edited the paper.

## FUNDING INFORMATION

This work was supported by MEXT KAKENHI grant numbers JP23K18432, JP22H03439, JP17K18040, JP25702041 to MM, and was supported by The Nakatomi Foundation, The Takeda Science Foundation, and The Hiroshima University Fund “Nozomi H Foundation” subsidy for the promotion of cancer treatment research. All funding agencies approved the broad elements of study design during the process of grant submission and review, and played no direct role in the design of the study and collection, analysis, and interpretation of data and in writing the manuscript.

## CONFLICT OF INTEREST STATEMENT

We have no conflict of interest to declare.

## ETHICS STATEMENT

All experimental procedures performed in this study were conducted in accordance with the institutional guidelines provided for the care and use of laboratory animals, which were approved by the Animal Ethics and Research Committee of the Health Sciences University of Hokkaido (no. 19‐060).

## Supporting information


Figure S1.

## Data Availability

All relevant data are within the manuscript.
