# Supplementary material for: Akt1 deficiency does not affect fiber type composition or mitochondrial protein expression in skeletal muscle of male mice
Source: Physiol Rep. 2024 Sep 10;12(17):e70048. doi: 10.14814/phy2.70048 (PMC11387151; doi:10.14814/phy2.70048)
Supplement: Supplementary file 1 — Figure S1. [file PHY2-12-e70048-s001.pdf]

Supplemental Figure 1

Original western blotting images & Detailed experimental conditions

The rectangle area with white dotted line was cropped and used for the figure.

pan-Akt (predicted size; 60 kDa)

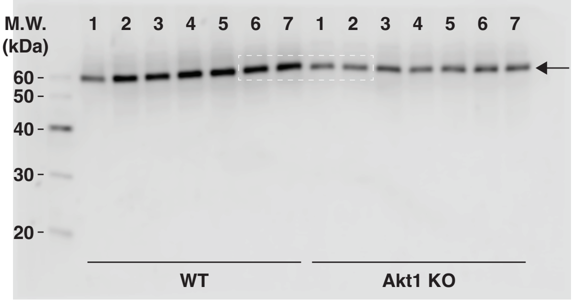

Protein Amount: 10 μg protein / lane

Gel system: TGX Any kD Precast Protein Gels, #4569036; BIO-RAD

Electrophoresis: 200V constant, 30-40 min

Transfer: Transblot Turbo; BIO-RAD, 2.5A contant, 3 min

Membrane: Trans-Blot® TurboTM RTA Transfer PVDF Kit

Blocking: Bullet Blocking One (13779-01; Nacalai Tesqu), 5 min at r.t.

Primary Antibody: Akt (pan) (C67E7) Rabbit mAb #4691; Cell Signaling Technology
1:1000 dilution, 4°C, Over Night

2nd Antibody: Peroxidase AffiniPure Goat Anti-Rabbit IgG (Cat#: 111-035-003)
Jackson ImmunoResearch, 1:15000 dilution, 60 min at r.t.

Detection: ImmunoStar Zeta, 291-72401, FUJIFILM Wako

Scan & Quantification: C-DiGit Blot Scanner; LI-COR Biosciences

Akt1 (predicted size; 60 kDa)

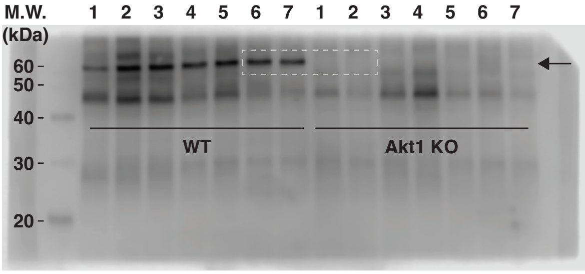

Protein Amount: 10 μg protein / lane

Gel system: TGX Any kD Precast Protein Gels, #4569036; BIO-RAD

Electrophoresis: 200V constant, 30-40 min

Transfer: Transblot Turbo; BIO-RAD, 2.5A contant, 3 min

Membrane: Trans-Blot® TurboTM RTA Transfer PVDF Kit

Blocking: Bullet Blocking One (13779-01; Nacalai Tesqu), 5 min at r.t.

Primary Antibody: Akt1 (2H10) Mouse mAb; Cell Signaling Technology
1:1000 dilution, 4°C, Over Night

2nd Antibody: Peroxidase AffiniPure Goat Anti-Anti-Mouse IgG (Cat#: 115-035-003)
Jackson ImmunoResearch, 1:15000 dilution, 60 min at r.t.

Detection: ImmunoStar Zeta, 291-72401, FUJIFILM Wako

Scan & Quantification: C-DiGit Blot Scanner; LI-COR Biosciences

Akt2 (predicted size; 60 kDa)

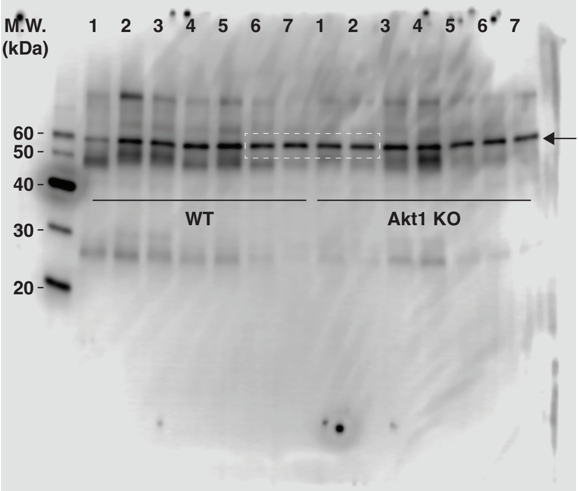

Protein Amount: 10 μg protein / lane

Gel system: TGX Any kD Precast Protein Gels, #4569036; BIO-RAD

Electrophoresis: 200V constant, 30-40 min

Transfer: Transblot Turbo; BIO-RAD, 2.5A contant, 3 min

Membrane: Trans-Blot® TurboTM RTA Transfer PVDF Kit

Blocking: Bullet Blocking One (13779-01; Nacalai Tesqu), 5 min at r.t.

Primary Antibody: Akt2 (5B5) Rabbit mAb; Cell Signaling Technology
1:1000 dilution, 4°C, Over Night

2nd Antibody: Peroxidase AffiniPure Goat Anti-Rabbit IgG (Cat#: 111-035-003)
Jackson ImmunoResearch, 1:15000 dilution, 60 min at r.t.

Detection: ImmunoStar Zeta, 291-72401, FUJIFILM Wako

Scan & Quantification: C-DiGit Blot Scanner; LI-COR Biosciences

Oxphos Complex

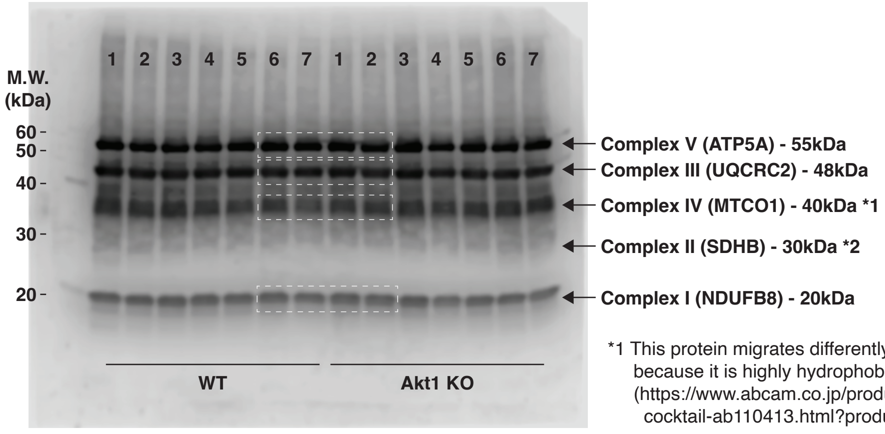

*1 This protein migrates differently in different gel systems, because it is highly hydrophobic. (https://www.abcam.co.jp/products/panels/total-oxphos-rodent-wb-antibody-cocktail-ab110413.html?productWallTab=ShowAll#lb)

*2 too faint for the quantification

Protein Amount: 10 μg protein / lane

Gel system: TGX Any kD Precast Protein Gels, #4569036; BIO-RAD

Electrophoresis: 200V constant, 30-40 min

Transfer: Transblot Turbo; BIO-RAD, 2.5A contant, 3 min

Membrane: Trans-Blot® TurboTM RTA Transfer PVDF Kit

Blocking: Bullet Blocking One (13779-01; Nacalai Tesqu), 5 min at r.t.

Primary Antibody: Total OXPHOS Rodent WB Antibody Cocktail ab110413; Abcam
1:1000 dilution, 4°C, Over Night

2nd Antibody: Peroxidase AffiniPure Goat Anti-Mouse IgG (Cat#: 115-035-003)
Jackson ImmunoResearch, 1:15000 dilution, 60 min at r.t.

Detection: ImmunoStar Zeta, 291-72401, FUJIFILM Wako

Scan & Quantification: C-DiGit Blot Scanner; LI-COR Biosciences

Complex II

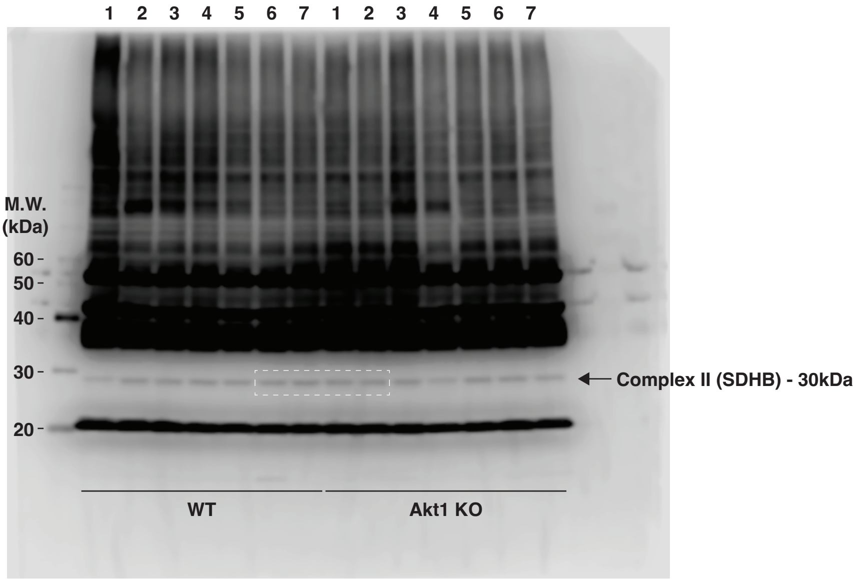

Protein Amount: 10 μg protein / lane

Gel system: e-PAGEL E-R520L (5～20%); ATTO

Electrophoresis: 20mA/Gel constant, 75-80 min

Transfer: Criterion Blotter; BIO-RAD, 0.3A contant, 60 min

Membrane: Immun-Blot PVDF; BIO-RAD

Blocking: Bullet Blocking One (13779-01; Nacalai Tesqu), 5 min at r.t.

Primary Antibody: Total OXPHOS Rodent WB Antibody Cocktail ab110413; Abcam
1:1000 dilution, 4°C, Over Night

2nd Antibody: Peroxidase AffiniPure Goat Anti-Mouse IgG (Cat#: 115-035-003)
Jackson ImmunoResearch, 1:15000 dilution, 60 min at r.t.

Detection: ImmunoStar LD, 296-69901, FUJIFILM Wako

Scan & Quantification: C-DiGit Blot Scanner; LI-COR Biosciences

PGC-1α (predicted size; 130 kDa)

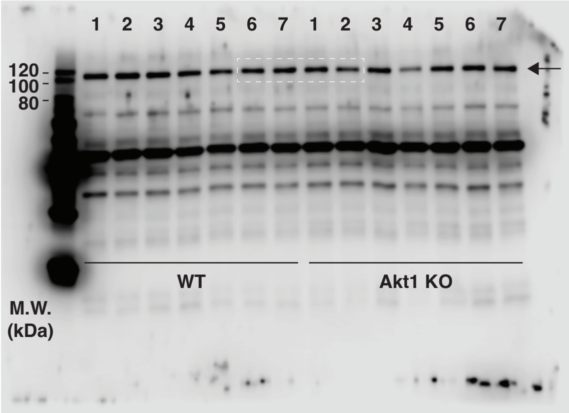

Protein Amount: 10 μg protein / lane

Gel system: TGX Any kD Precast Protein Gels, #4569036; BIO-RAD

Electrophoresis: 200V constant, 30-40 min

Transfer: Transblot Turbo; BIO-RAD, 2.5A contant, 3 min

Membrane: Trans-Blot® TurboTM RTA Transfer PVDF Kit

Blocking: Bullet Blocking One (13779-01; Nacalai Tesqu), 5 min at r.t.

Primary Antibody: Anti-PGC-1 Antibody AB3242; Merck Millipore
1:1000 dilution, 4°C, Over Night

2nd Antibody: Peroxidase AffiniPure Goat Anti-Rabbit IgG (Cat#: 111-035-003)
Jackson ImmunoResearch, 1:15000 dilution, 60 min at r.t.

Detection: ImmunoStar Zeta, 291-72401, FUJIFILM Wako

Scan & Quantification: C-DiGit Blot Scanner; LI-COR Biosciences

Phospho-ACLY-S455 (predicted size; 121 kDa)

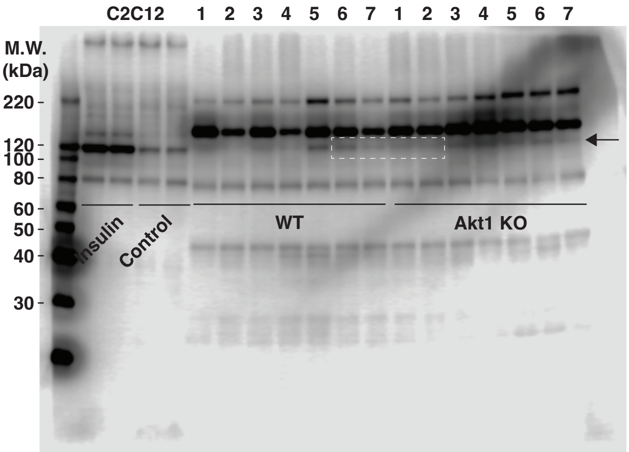

Protein Amount: 10 μg protein / lane

Gel system: e-PAGEL E-R520L (5～20%); ATTO

Electrophoresis: 20mA/Gel constant, 75-80 min

Transfer: Criterion Blotter; BIO-RAD, 0.3A contant, 120-150 min

Membrane: Immun-Blot PVDF; BIO-RAD

Blocking: Bullet Blocking One (13779-01; Nacalai Tesqu), 5 min at r.t.

Primary Antibody: Phospho-ACLY-S455 Rabbit pAb AP0779; ABclonal
1:1000 dilution, 4°C, Over Night

2nd Antibody: Peroxidase AffiniPure Goat Anti-Rabbit IgG (Cat#: 111-035-003)
Jackson ImmunoResearch, 1:15000 dilution, 60 min at r.t.

Detection: ImmunoStar LD, 296-69901, FUJIFILM Wako

Scan & Quantification: C-DiGit Blot Scanner; LI-COR Biosciences

A protein lysate prepared from untreated and insulin-treated murine myoblasts (C2C12) was included as a positive control for the phosphorylation of ACLY protein.
C2C12 myoblasts were treated with insulin (10nM) dissolved in serum/antibiotics-free media for 15 min.

Total-ACLY (predicted size; 121 kDa)

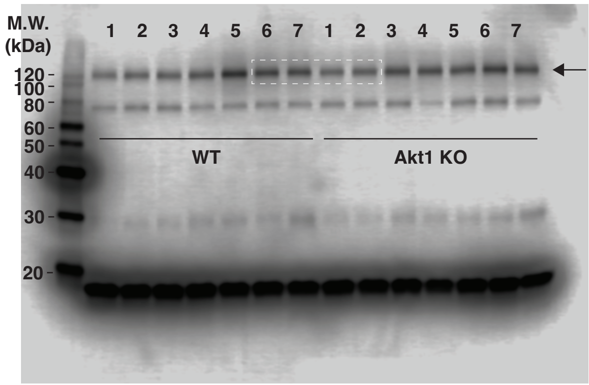

Protein Amount: 10 μg protein / lane

Gel system: TGX Any kD Precast Protein Gels, #4569036; BIO-RAD

Electrophoresis: 200V constant, 30-40 min

Transfer: Transblot Turbo; BIO-RAD, 2.5A contant, 3 min

Membrane: Trans-Blot® TurboTM RTA Transfer PVDF Kit

Blocking: Bullet Blocking One (13779-01; Nacalai Tesqu), 5 min at r.t.

Primary Antibody: ATP-Citrate Lyase (D1X6P) Rabbit mAb #13390; CST
1:1000 dilution, 4°C, Over Night

2nd Antibody: Peroxidase AffiniPure Goat Anti-Rabbit IgG (Cat#: 111-035-003)
Jackson ImmunoResearch, 1:15000 dilution, 60 min at r.t.

Detection: ImmunoStar LD, 296-69901, FUJIFILM Wako

Scan & Quantification: C-DiGit Blot Scanner; LI-COR Biosciences

GLUT4 (predicted size; 55 kDa)

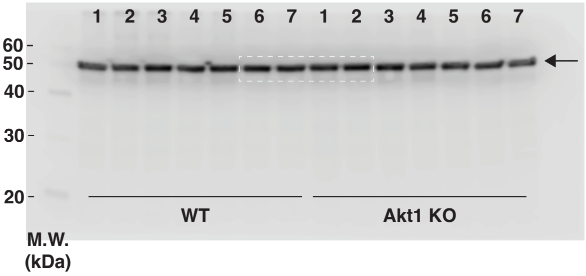

Protein Amount: 10 μg protein / lane

Gel system: TGX Any kD Precast Protein Gels, #4569036; BIO-RAD

Electrophoresis: 200V constant, 30-40 min

Transfer: Transblot Turbo; BIO-RAD, 2.5A contant, 3 min

Membrane: Trans-Blot® TurboTM RTA Transfer PVDF Kit

Blocking: Bullet Blocking One (13779-01; Nacalai Tesqu), 5 min at r.t.

Primary Antibody: GLUT4 Monoclonal antibody 66846-1-Ig; Proteintech
1:2000 dilution, 4°C, Over Night

2nd Antibody: Peroxidase AffiniPure Goat Anti-Anti-Mouse IgG (Cat#: 115-035-003)
Jackson ImmunoResearch, 1:15000 dilution, 60 min at r.t.

Detection: ImmunoStar Zeta, 291-72401, FUJIFILM Wako

Scan & Quantification: C-DiGit Blot Scanner; LI-COR Biosciences

Phos-FoxO1-Thr24 (predicted size; 78-82 kDa)

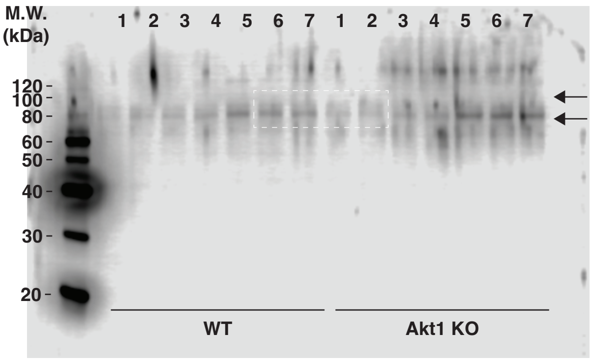

phos-FoxO3a (Thr32), 95 kDa, undetermined

phos-FoxO1 (Thr24), 78-82 kDa

Protein Amount: 10 μg protein / lane

Gel system: TGX Any kD Precast Protein Gels, #4569036; BIO-RAD

Electrophoresis: 200V constant, 30-40 min

Transfer: Transblot Turbo; BIO-RAD, 2.5A contant, 3 min

Membrane: Trans-Blot® TurboTM RTA Transfer PVDF Kit

Blocking: Bullet Blocking One (13779-01; Nacalai Tesqu), 5 min at r.t.

Primary Antibody: Phospho-FoxO1 (Thr24)/FoxO3a (Thr32) Antibody
#9464; CST
1:1000 dilution, 4°C, Over Night

*Phospho-Fox01 (Thr24)/Fox03a (Thr32) Antibody detects endogenous levels of Fox01/Fox03a only when phosphorylated at threonine 24 of Fox01 or threonine 32 of Fox03a.

2nd Antibody: Peroxidase AffiniPure Goat Anti-Rabbit IgG (Cat#: 111-035-003)
Jackson ImmunoResearch, 1:15000 dilution, 60 min at r.t.

Detection: ImmunoStar Zeta, 291-72401, FUJIFILM Wako

Scan & Quantification: C-DiGit Blot Scanner; LI-COR Biosciences

Total-FoxO1 (predicted size; 78-82 kDa)

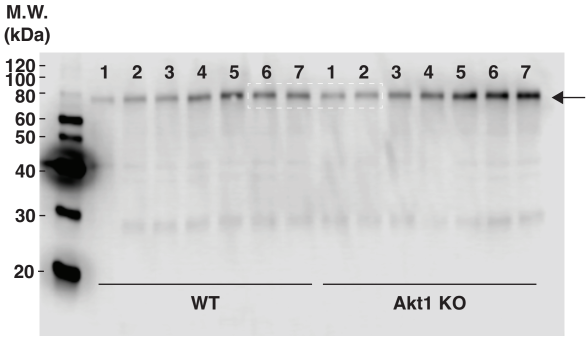

Protein Amount: 10 μg protein / lane

Gel system: TGX Any kD Precast Protein Gels, #4569036; BIO-RAD

Electrophoresis: 200V constant, 30-40 min

Transfer: Transblot Turbo; BIO-RAD, 2.5A contant, 3 min

Membrane: Trans-Blot® TurboTM RTA Transfer PVDF Kit

Blocking: Bullet Blocking One (13779-01; Nacalai Tesqu), 5 min at r.t.

Primary Antibody: FoxO1 (C29H4) Rabbit mAb #2880; CST
1:1000 dilution, 4°C, Over Night

2nd Antibody: Peroxidase AffiniPure Goat Anti-Rabbit IgG (Cat#: 111-035-003)
Jackson ImmunoResearch, 1:15000 dilution, 60 min at r.t.

Detection: ImmunoStar Zeta, 291-72401, FUJIFILM Wako

Scan & Quantification: C-DiGit Blot Scanner; LI-COR Biosciences
